# COVID-19 and Bradyarrhythmias: A Case Report With Literature Review

**DOI:** 10.7759/cureus.21552

**Published:** 2022-01-24

**Authors:** Saad Ali Ansari, Yusra Ansari, Tahir Muhammad Abdullah Khan

**Affiliations:** 1 Internal Medicine, University of California Riverside School of Medicine, Riverside, USA; 2 Medicine, University of Kentucky, Bowling Green, USA; 3 Internal Medicine, Rawalpindi Medical University, Rawalpindi, PAK; 4 Pulmonary and Critical Care Medicine, University of Kentucky, Bowling Green, USA

**Keywords:** complete heart block, pacemaker, bradyarrhythmia, sinus arrest, sinus pauses, covid-19

## Abstract

We report a case of a 51-year-old male with no past medical history who was admitted for acute hypoxic respiratory failure secondary to COVID-19. During his hospitalization, the patient developed sinus bradycardia and frequent sinus pauses were observed on telemetry. No other cause of his bradyarrhythmia was identified except for his COVID-19 infection. There has been numerous case reports and case series describing different arrhythmias seen in patients infected with COVID-19. We present a case of sinus arrest in a patient with COVID-19 and a review of other case reports describing bradyarrhythmia in COVID-19 patients.

## Introduction

The COVID-19 pandemic due to severe acute respiratory syndrome coronavirus 2 (SARS-CoV-2) has affected millions of people worldwide since the emergence of the first case in Wuhan, China, in December 2019. Severe acute respiratory syndrome coronavirus 2 has spike (S) proteins on its surface which are responsible for binding to host cell receptors and fusion of viral and cellular membranes. Angiotensin-converting enzyme 2 (ACE 2) is identified as a functional receptor for the spike protein of SARS-CoV-2. The ACE 2 is expressed in high concentrations in the lung (type II alveolar cells), heart, esophagus, ilium, kidney, and urinary bladder [1,2]. Patients infected with SARS-CoV-2 manifest symptoms ranging from mild to severe respiratory disease including acute respiratory distress syndrome (ARDS) and can involve multiorgan failure.

Although the respiratory system is the most affected organ system, other organ systems involvement has been frequently reported which may manifest as encephalopathy, a hypercoagulable state with thromboembolic disease, deranged liver enzymes, acute kidney failure, and myocardial injury (myocarditis). Cardiac injury (defined as an increase in high sensitivity troponins T or I level more than the 99th percentile upper reference limit and new ECG or transthoracic echocardiographic changes) has been reported in 49% of patients with critical illness secondary to COVID-19. The most frequent abnormalities observed were EKG or echocardiographic signs of left ventricle abnormalities (87%), and right ventricular dysfunction (47%) which was more common than left ventricular dysfunction (13%). Other abnormalities observed were pericardial effusion (43%), new-onset atrial arrhythmias (33%), left ventricular relaxation impairment (33%), and left ventricle (LV) systolic dysfunction (13%) [3]. Among conduction abnormalities, sinus node dysfunction with sinus arrest secondary to COVID-19 is rarely reported in the literature and prognosis and disease course in these patients is unknown yet. We report a case of sinus arrest secondary to COVID-19 infection in a critically ill patient who required a permanent pacemaker.

## Case presentation

A 51-year-old Caucasian male patient, active cigarette smoker, unvaccinated for COVID-19, and no prior history of any medical illness, presented with worsening shortness of breath. The patient tested positive for COVID-19 a few days prior to presentation. On presentation, patient had a blood pressure of 137/80 mm Hg, heart rate of 93 beats per minute, he was afebrile and was saturating at 92 % on 15 L of O2 via a non-rebreather mask. He was alert and awake but was showing signs of respiratory distress using accessory respiratory muscle and was tachypneic. Chest auscultation showed bilateral lower lung crackles with no wheezing. Laboratory tests of the patient are given in Table 1.

**Table 1:** Laboratory work-up

Laboratory Parameter	Patient’s Results	Normal Range
White blood count	24.1 k/uL	4-11 K/uL
Hemoglobin	15.3 g/dl	13-18 g/dl
Platelet	344 K/uL	140-440 K/uL
Serum creatinine	1.34 mg/dl	0.66-1.25 mg/dl
Serum bicarbonate	24 mmol/L	22-30 mmol/L
Lactic acid	5.9 mmol/L	0.7-2.0 mmol/l
Alanine aminotransferase	70 U/L	0-49 U/L
Aspartate aminotransferase	112 U/L	17-36 U/L
Total bilirubin	1.2 mg/dl	0.2-1.0 mg/dl
C-reactive protein (CRP)	18.9 mg/dl	0-1.0 mg/dl
Procalcitonin	0.6 ng/mL	< 0.15 ng/mL

**Figure 1:**
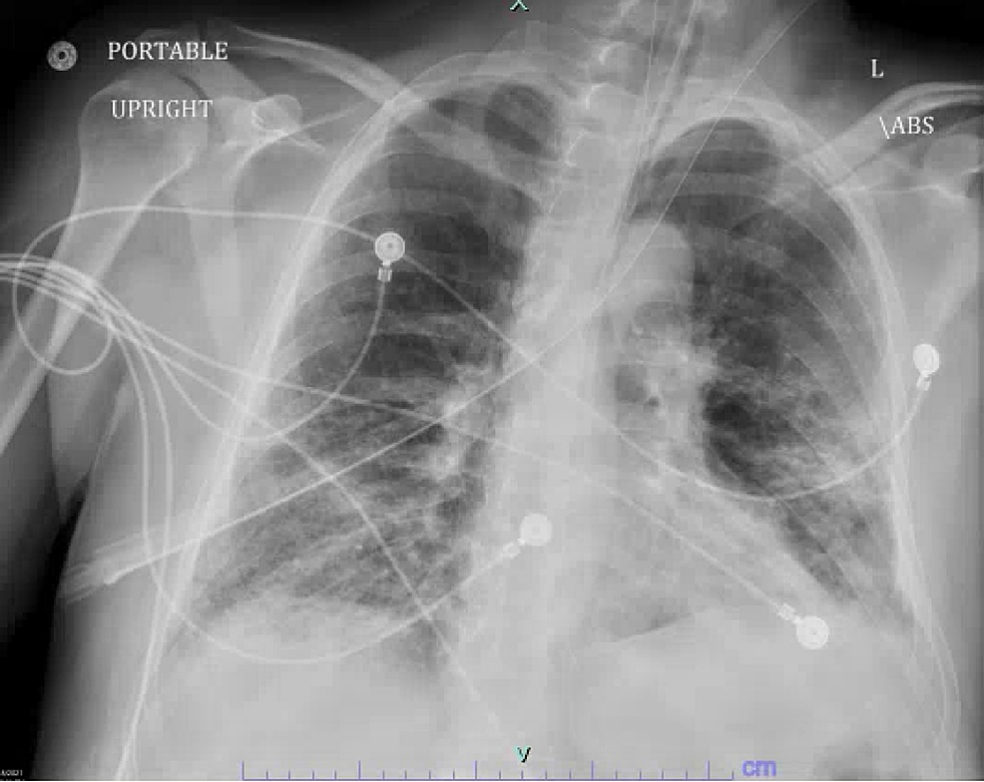
Chest X-ray on initial presentation showing bilateral diffuse infiltrates consistent with COVID-19

**Figure 2:**
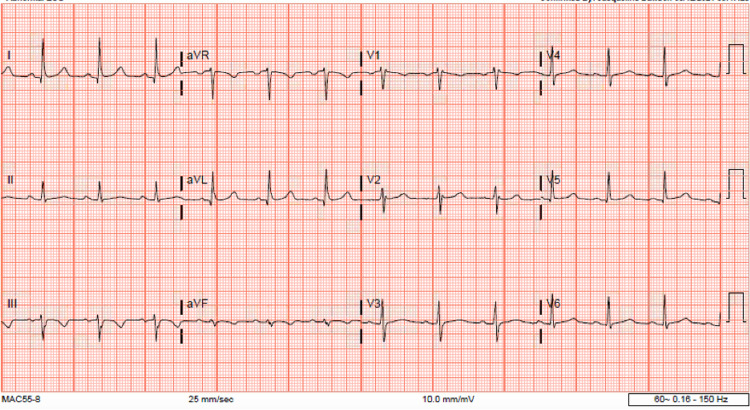
EKG on initial presentation

Chest X-ray (CXR) showed bilateral ground-glass infiltrates consistent with COVID -19 pneumonia and left pleural effusion (Figure 1). The EKG showed no acute ischemic changes, PR interval at 184 milliseconds (ms), QTc of 490 ms ( Figure 2) and troponin were elevated to 0.10 ug/mL (0-0.034 ug/mL) which subsequently trended down to normal values.

Patient developed respiratory distress in the emergency room and arterial blood gas (ABG) showed PH 7.33, partial pressure of carbon dioxide (PaCO2) at 51, partial pressure of oxygen (PaO2) at 56, on 100% fraction of inspired oxygen (FiO2). The patient was intubated and supported with lung protective strategy of mechanical ventilation. Deep tracheal aspirates were sent for gram stain and culture, and BioFire® FilmArray® (BioFire Inc., Salt Lake City, UT, USA) pneumonia (PN) panel testing post which the patient was initiated on dexamethasone 10 mg twice a day and broad-spectrum antibiotics with linezolid and cefepime. When the sputum culture and pneumonia polymerase chain reaction (PCR) panel did not suggest bacterial infection, the patient was given one dose of 400 mg of sarilumab, and antibiotics were discontinued. Intravenous (iv) propofol and fentanyl infusions were used for sedation and analgesia, and he required a low dose of norepinephrine for sedation induced mild hypotension. Proning protocol was implemented. 

**Figure 3:**
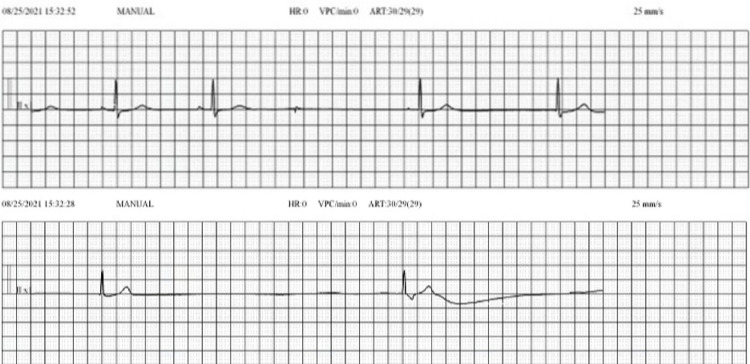
Sinus bradycardia with sinus pauses on Telemetry Telemetry strips showing sinus pauses of approximately 2.2 and 3.6 s.

Over the following week, the patient developed sinus bradycardia necessitating change of sedatives from propofol to midazolam, and intermittent iv fentanyl for adequate analgesia. Two weeks into his admission, patient developed ventilator associated bacterial pneumonia (VAP) with pan sensitive *Escherichia coli *which was further complicated by left side pneumothorax warranting placement of 14 French-size pigtail catheters and iv ceftriaxone for VAP. Unfortunately, the patient developed worsening sinus bradycardia with heart rate as low as 27 with frequent sinus pauses observed on telemetry (Figure 3) with largest pause of 12 seconds along with loss of arterial pulse waveform. A transvenous pacemaker was placed. 

**Figure 4:**
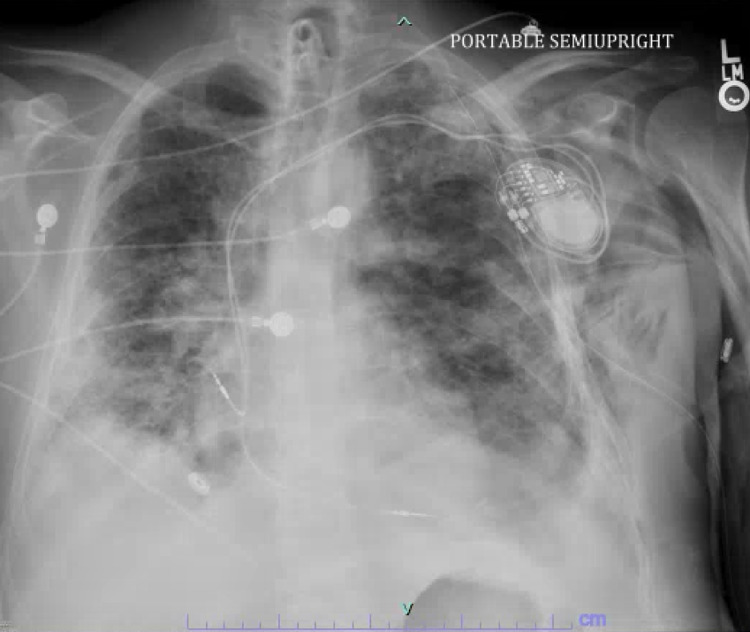
Chest X-ray after pacemaker placement

The EKG showed mild LV hypertrophy with LV ejection fraction of 60% to 65% and no regional wall motion abnormality. After treatment of VAP, requirement of supplemental oxygen improved but he could not be liberated from mechanical ventilator due to excessive respiratory secretions. Bedside percutaneous tracheostomy was performed without complication, and due to persistent intermittent dependence of transvenous pacemaker, a permanent pacemaker (Figure 4) was placed without complication as per discretion of electrophysiologist. The patient was later transferred to long term acute care facility (LTAC) for weaning from ventilator and physical and occupational therapy. Unfortunately, the patient's condition was complicated by bilateral lower extremities deep venous thrombosis requiring anticoagulation therapy and later by the second event of ventilator associated pneumonia with *Stenotrophomonas maltophilia* with bacteremia and septic shock. The patient’s family opted for comfort care and he succumbed to his illness.

## Discussion

We reviewed a total of 30 case reports/case series involving a total of 67 patients utilizing advanced search on PubMed. Keywords used for literature review were: "COVID-19", "bradyarrhythmias", "bradycardia", "sinus arrest", "sinus pauses", "atrioventricular (AV) block", "heart block", "complete heart block (CHB)", "sinus node dysfunction". Case reports from the start of the pandemic till January 2022 were reviewed. Out of a total of 67 patients, 54% were males (36/67) while 46% were females (31/67). The mean age of patients was 56.3 ± 15.2, mean age for male patients was 53.29 ± 15.29 and mean age of female patients was 60.4 ± 14.1. Data regarding comorbidities was available for 65/67 patients, 60% (39/65) of patients were found to have one or more cardiac risk factors. Baseline EKG information was provided for 41 patients, out of them 71% (29/41) had normal baseline EKG. One important observation was that only a very small percentage of patients who developed bradyarrhythmias eventually required intubation and ventilatory support. Of the 53 patients with available data regarding intubation, only 19% (13/67) were put on ventilatory support with one patient declining intubation. Data regarding troponin level and ECHO reading were available for 93% (62/67) patients. Only 16% (10/67) had some degree of troponin elevation and only 11% (7/62) had a left ventricular ejection fraction (LVEF) < 50% on ECHO. Many of the patients experienced more than one type of bradyarrhythmia during their clinical course. The most common bradyarrhythmia among the patients was complete heart block (CHB) in 51% of patients (34/67) followed by sinus bradycardia in 30% (20/67), sinus arrest in 17.9% (12/67), second-degree atrioventricular block (AV) block in 7% (5/67) and high AV block in 3% (2/67). Only 30% (20/67) of patients eventually required a permanent pacemaker while one patient declined permanent pacemaker placement. Around 18 % required temporary pacing. The majority of patients i.e., 52% (35/67) did not require any type of pacing either permanent or temporary and experienced only transient bradyarrhythmia which resolved on its own. Mortality was reported in only 15% (10/67) of the patients. Table 2 summarizes the data described above.

**Table 2:** Table comprising data compiled from published case reports/case series on bradyarrhythmia's seen in patients with COVID-19 infection. n/a: Data not available, HTN: Hypertension, DM: Diabetes mellitus, HLD: Hyperlipidemia, CVA: Cerebrovascular accident, LVEF: Left ventricular ejection fraction, CHB: Complete heart block, PPM: Permanent pacemaker, AV: Atrioventricular, CAD: Coronary artery disease, AS: Aortic stenosis, BMI: Body mass index, CMP: Cardiomyopathy, COPD: Chronic obstructive pulmonary disease, CHF: Congestive heart failure, MI: Myocardial infarction, OSA: Obstructive sleep apnea, CVD: Cardiovascular diseases, aVL: Augmented vector left, RBBB: Right bundle branch block, LAFB: Left anterior fascicular block, LPFB: Left posterior fascicular block, LAHB: Left anterior hemiblock

Reference No.	Age	Gender	Comorbidities	LVEF and Troponin	Intubation	Day since admission when EKG changes noted	Baseline EKG on Admission	EKG Changes	Pacemaker	Outcome
[4]	69	Female	HTN, DM, CVA, Asthma, HLD	Normal	No	8	Normal	2:1 AV Block, sinus arrest	No	Discharged
[4]	83	Female	HTN, HLD	Normal LVEF, Elevated Trop	No	8	Normal	Sinus Arrest	No	Discharged
[5] (Case series of 7 Patients)	n/a	Males 3/7 Females 4/7	HTN 5/7, HLD 1/7, DM 6/7 Hepatic Disease/Cirrhosis 2/7 CVD 1/7, CKD 1/7	Normal LVEF in all patients Troponin elevated in 2/7	n/a	n/a	First Degree AV Block 1/7 RBBB 2/7, LAHB+RBBB 1/7	CHB 2/7, Sinus Bradycardia and CHB with 1/7, 2:1 AV block 1/7, Sinus pauses/sinus arrest 3/7	Temporary Pacemaker 3/7 Permanent Pacemaker 4/7	Patients died 5/7 Discharged 2/7
[6]	34	Male	Bipolar Disorder, Hypothyroidism	Normal	Yes	n/a	Normal	Sick sinus syndrome	Permanent Pacemaker	Discharged
[7]	70	Female	None	Normal	Yes	2	Normal	Sinus Bradycardia	No	Discharged
[7]	81	Male	Ascending Aortic Aneurysm, OSA HTN	Normal LVEF, Elevated Troponin	Yes	4	Normal	Sinus Bradycardia	No	Discharged
[8]	67	Male	HTN, HLD, Ex-Smoker	Normal LVEF, Elevated Troponin	Yes	7	Sinus bradycardia with first degree AV Block	Sinus pauses	Transcutaneous pacing only	Discharged
[9] (Case series of 7 Patients)	n/a	Males 4/7 Females 3/7	DM, HTN, MI 2/7 CHF 1/7	Normal ECHO 5/7, LVEF < 50 2/7 Elevated Troponin 1/7	n/a	1	n/a	CHB 5/7 Sick sinus syndrome 2/7	PPM in 5 patients with CHB	Discharged
[10]	54	Male	n/a	Normal LVEF	Yes	14	Normal	CHB	No	Death while hospitalized
[11]	58	Female	HTN, DM, CHF	LVEF 45-50 %, Normal Trop	No	5	Normal	Sinus bradycardia	Permanent Pacemaker	Discharged
[12]	72	Female	HTN, DM	Normal LVEF, Elevated Troponin	Yes	n/a	n/a	Sinus bradycardia CHB, Pulseless arrest	Temporary Pacemaker	Discharged
[13]	47	Female	None	Normal	No	n/a	n/a	Sinus bradycardia, Sinus pauses Junctional escape rhythm with AV dissociation	No	Not admitted in hospital
[14]	42	Female	DM, BMI > 30	Normal LVEF, Elevated Trop	No	2	n/a	CHB	No	Discharged
[14]	62	Male	HTN, DM, CAD, BMI > 25	Normal LVEF, Elevated Trop	No	1	n/a	CHB	Temporary Pacemaker	Discharged
[14]	61	Male	HTN, BMI > 25	Normal	No	2	n/a	CHB	No	Discharged
[14]	64	Male	HTN, BMI > 25	Normal	No	2	n/a	CHB	No	Discharged
[15]	75	Female	None	Normal	Yes	33	n/a	Sinus bradycardia, Sinus pauses	Permanent Pacemaker	Discharged
[16]	55	Female	None	Normal	No	2	Normal	Sinus bradycardia, Sinus pauses	No	Discharged
[17]	50	Female	BMI > 30	Normal	No	n/a	n/a	Sinus bradycardia	Permanent Pacemaker	Discharged
[17]	65	Female	HTN, DM	Normal	No	n/a	n/a	CHB	Permanent Pacemaker	Discharged
[17]	43	Female	None	Normal	No	n/a	n/a	CHB	Temporary Pacemaker	Discharged
[17]	25	Male	None	Normal	No	n/a	n/a	CHB	No	Discharged
[17]	60	Male	HTN	Normal	No	n/a	n/a	CHB	No	Discharged
[17]	70	Female	HTN	Normal	No	n/a	n/a	CHB	Permanent Pacemaker	Discharged
[18]	71	Female	Parkinson Disease, Tardive Dyskinesia DM, Bipolar Disorder	Normal	No	n/a	n/a	CHB	Permanent Pacemaker	Discharged
[19]	38	Female	None	Normal	No	n/a	n/a	Sinus bradycardia, CHB	No	Discharged
[20]	48	Male	None	Normal	No	1	n/a	CHB	No	Discharged
[21]	36	Male	None	Normal	No	n/a	n/a	Sinus node dysfunction, sinus pauses/ sinus node arrest	Permanent Pacemaker	Discharged
[22]	44	Male	DM	Normal	No	1	n/a	CHB	No	Discharged
[23] (Case series of 6 Patients)	n/a	Male – 3/6 Female – 3/6	HTN – 5/6	Normal LVEF – 5/6, EF – 45-50 % - 1/6, Normal Trop – 6/6	No	6, 4, 3, 4, 3, 3	Normal 6/6	Sinus Bradycardia 6/6	No	Discharged
[24]	41	Male	Familial Mediterranean Fever	Normal	Yes	5	Normal	CHB	No	Death while hospitalized
[24]	77	Male	None	Normal	Yes	7	Normal	CHB, sinus pause	Temporary Pacemaker	Death while hospitalized
[24]	36	Female	n/a	LVEF 30 %, Normal Troponin	Yes	5	Sinus Tachycardia	CHB	No	Discharged
[25]	60	Male	HTN, Non-Ischemic Dilated CMP, COPD HLD, Cocaine abuse	LVEF-25 %, Normal Troponin	No	1	LBBB	High Grade AV Block w RBBB escape morphology	No	Discharged
[26]	49	Male	HTN	Normal	No	n/a	n/a	CHB	Permanent Pacemaker	Discharged
[27]	41	Male	DM	Normal	No	2	Normal	Paroxysmal AV block, Sinus arrest	Permanent Pacemaker	Discharged
[28]	53	Male	None	Mildly impaired LVEF, Normal Troponin	No	7	Normal	Type 2 AV block (Mobitz 2), CHB, High Degree AV Block	Permanent Pacemaker	Discharged
[29]	23	Male	Stage 3b Hodgkin’s Lymphoma	LVEF 35-40 %, Elevated Troponin	No	1	Sinus Tachycardia with RBBB	CHB	Temporary Pacemaker	Discharged
[30]	74	Female	DM	Normal Troponin	Declined by patient	n/a	RBBB w LAFB	RBBB w LPFB, 2^nd^ Degree AV Block (Mobitz Type 2)	Declined by patient	Death while hospitalized
[31]	82	Male	HTN, CVA	Normal Troponin	Yes	1	Normal	CHB	No	Death while hospitalized
[31]	55	Male	None	Normal Troponin initially later becoming Elevated, RH strain of bedside ECHO	Yes	6	RBBB, ST depressions at inferior leads Present at baseline	2:1 AV Block	No	Discharged
[31]	43	Male	None	Normal	Yes	24	Normal	CHB	No	Discharged
[32]	56	Male	DM	Normal	No	3	Normal	CHB	Temporary pacemaker	Discharged
[32]	48	Male	HTN	Normal	No	5	T wave inversions V1-V2	CHB	No	Discharged
[32]	57	Female	None	Normal	No	n/a	Normal	CHB	Temporary Pacemaker	Discharged
[32]	42	Female	DM	Normal	No	3	T wave inversions lead 1, aVl and V2	CHB	Temporary Pacemaker	Discharged
[33]	55	Male	Hypothyroidism, BMI > 30	n/a	No	1	Normal	Sinus Bradycardia	No	Discharged
[33]	60	Female	None	n/a	No	1	Normal	Sinus Bradycardia	No	Discharged
[33]	78	Female	Hypothyroidism, CAD, HTN, HLD, BMI > 25	n/a	No	4	Normal	Sinus Bradycardia	No	Discharged
[33]	73	Male	CAD, HTN, HLD, AS, BMI >35	n/a	No	4	Normal	Sinus Bradycardia	No	Discharged

We presented a case of a patient without any major comorbidities who was admitted to the hospital for COVID-19 pneumonia and later during hospitalisation developed sinus bradycardia with frequent sinus pauses for which a permanent pacemaker had to be placed. In our patient who had no significant cardiac risk factors, sinus bradycardia and pauses are possibly related to conduction abnormalities caused by COVID-19. COVID-19 has been associated with myocarditis, myocardial infarction, hypercoagulable state, arrhythmias, and conduction abnormalities [34]. From our literature review, it is evident that a lot of patients with COVID-19 who develop bradyarrhythmia have little or no cardiac risk factors. The extent of respiratory compromise or critical illness does not always correlate with conduction abnormalities as only 19% of patients who developed bradyarrhythmia in our review were intubated and put on ventilatory support. Long term effects of COVID-19 on the conduction system is still to be studied. In the majority of patients, only transient bradycardia or conduction abnormalities were noted. However, up to 30% of patients did end up getting permanent pacemaker placement. Outpatient follow-ups of patients who develop arrhythmias while hospitalized for COVID-19 infection is necessary to study the long term effects on the cardiac conduction system.

## Conclusions

In conclusion, COVID-19 is associated with wide-ranging cardiac manifestations including bradyarrhythmias. These bradyarrhythmias do not always correlate with severe COVID-19 infection and can present in individuals without any cardiac risk factors. In the majority of patients, these are only transient and resolve as COVID-19 infection subsides, but further study is required to determine the long-term effects of COVID-19 on the conduction system of the heart.
